# Same dose, different impact: acute cannabis intoxication impairs visuospatial working memory in both sexes, with disproportionate male vulnerability

**DOI:** 10.3389/fnbeh.2026.1785335

**Published:** 2026-04-16

**Authors:** Chen Hanna Ryder, Carmit Gal, Einav Levy, Yifaat Tamarkin Leider, Tal Katzenelson, Tomer Vainshtein, Mohammad Ebrahim Naffaa, Samih Badarny, Yazid Badarny

**Affiliations:** 1 The Brain and Behavior Research Institute, Western Galilee College, Acre, Israel; 2 Department of Social Work, Research Center for Innovation in Social Work, Tel-Hai University of Kiryat Shmona in the Galilee, Kiryat Shmona, Israel; 3 Department of Criminology and Law Enforcement, Beit Berl College, Kfar Saba, Israel; 4 Rheumatology Unit, Galilee Medical Center, Nahariya, Israel; 5 Azrieli Faculty of Medicine, Bar-Ilan University, Safed, Israel; 6 Department of Neurology, Galilee Medical Center, Nahariya, Israel; 7 Department of Neurosurgery, Rambam Medical Center, Haifa, Israel

**Keywords:** acute intoxication, cannabis, cognitive selectivity, domain-specific impairment, fronto-parietal network, precision medicine, sex differences, visuospatial working memory

## Abstract

**Introduction:**

With expanding global cannabis legalization and rising usage rates, elucidating the specific neurocognitive impact of acute cannabis intoxication across biological sexes is critical.

**Methods:**

Using a 2 × 2 factorial design, we examined 154 adults: 77 individuals who use cannabis regularly (≥ 5 days/week for ≥ 1 year; 46 males, 31 females) and 77 matched controls (32 males, 45 females). Participants completed standardized Wechsler Memory Scale subtests assessing four distinct memory domains during the peak pharmacokinetic window (45 min post-consumption of medical-grade cannabis: 16.1% THC, < 1% CBD).

**Results:**

Results demonstrated notable neuropsychological specificity: visuospatial working memory was selectively impaired, whereas auditory-verbal and short-term memory domains remained completely intact—a pattern strongly implicating disruption of fronto-parietal networks rich in CB1 receptors. Crucially, a significant Group × Sex interaction, *F*(1, 150) = 9.74, *p* < 0.01, η*p*^2^ = 0.061, revealed differential vulnerability: males exhibited a disproportionately larger deficit relative to male controls (Cohen’s *d* = −0.87, *p* < 0.001)—nearly double the impairment magnitude observed in females (*d* = −0.48, *p* < 0.05).

**Discussion:**

These findings advance our understanding of cannabis neuropharmacology by demonstrating that cognitive vulnerability is both domain-specific and sex-dependent, with direct implications for precision medicine approaches to cannabis therapeutics and sex-informed harm reduction strategies.

## Introduction

### The neurocognitive effects of cannabis

The evolving legal landscape and increasing societal acceptance of cannabis have led to unprecedented global usage rates, creating an urgent need to elucidate its neurocognitive consequences with greater precision (Hurd, 2020; Kroon et al., 2022; Le Foll et al., 2024). Early research often characterized the effects of cannabis monolithically. However, accumulating evidence indicates that its neurobiological impact is a complex, multifactorial phenomenon modulated by variables including Δ9-tetrahydrocannabinol (THC) concentration, consumption patterns, and individual neurobiological characteristics (Iacono et al., 2017; Le Foll et al., 2024; Scott et al., 2018). This scientific shift occurs as public perception may underestimate the potential neurocognitive risks of cannabis, creating a “perception gap” that rigorous research must address to inform policy and personal decision-making (Hurd, 2020; Prieto-Arenas et al., 2022).

Working memory, the limited-capacity system for temporarily maintaining and manipulating information, is fundamental to higher-order cognition including reasoning and learning and represents a particularly sensitive domain for investigating cannabis effects (Baddeley, 2012; Crean et al., 2011). Unlike previous research that has often focused on residual effects observed after periods of abstinence (Bourque and Potvin, 2021), the current investigation specifically targets the acute intoxicated state superimposed on a background of regular use. This distinction is vital, as the acute phase represents the functional state in which potential errors in judgment and performance decrements occur in real-time scenarios such as driving or operating machinery. Systematic reviews and meta-analyses consistently demonstrate that cannabis use is associated with small-to-moderate deficits across multiple cognitive domains, with verbal learning, memory, and executive functions being particularly vulnerable (Dellazizzo et al., 2022; Schoeler and Bhattacharyya, 2013). The primary psychoactive constituent, THC, mediates these effects through partial agonism at cannabinoid type 1 (CB1) receptors, which are densely distributed in the prefrontal cortex and hippocampus (Bloomfield et al., 2019; Le Foll et al., 2024).

From a cognitive psychology perspective, working memory represents a dynamic interface between perception, long-term memory, and action, enabling the temporary storage and manipulation of information necessary for complex cognitive tasks (Cowan, 2017; Oberauer, 2019). This cognitive system is capacity-limited, typically maintaining 3–5 chunks of information in an activated state, and relies on both domain-general executive processes and domain-specific

storage systems (Cowan, 2017). Systematic reviews and meta-analyses consistently demonstrate that cannabis use is associated with small-to-moderate deficits across multiple cognitive domains, with verbal learning, memory, and executive functions being particularly vulnerable (Dellazizzo

These impairments are not confined to periods of acute intoxication but can manifest as residual effects detectable for several days following use, although they diminish significantly with longer periods of abstinence (Crean et al., 2011; Scott et al., 2018). The primary psychoactive constituent, THC, mediates these effects through its action as a partial agonist at cannabinoid type 1 (CB1) receptors. These receptors are densely distributed in brain regions integral to cognition, including the prefrontal cortex and hippocampus, thereby disrupting the neural circuits that underpin

### The multicomponent architecture of working memory

Contemporary cognitive neuroscience conceptualizes working memory not as a unitary construct but as a multicomponent system. The influential Baddeley-Hitch model delineates distinct subsystems, including the phonological loop for auditory-verbal information and the visuospatial sketchpad for visual-spatial information (Baddeley, 2000; Baddeley and Hitch, 1974).

Beyond this classical model, contemporary theories emphasize additional dimensions of working memory organization. The embedded-processes model (Cowan, 1999) proposes that working memory emerges from the interaction between activated long-term memory representations and a focus of attention with limited capacity. Furthermore, recent evidence supports a distinction between maintenance (passive storage) and manipulation (active processing) components, with the

Individual differences in working memory capacity have been shown to predict performance on a wide range of higher-order cognitive tasks, including reading comprehension, mathematical problem-solving, and fluid intelligence (Engle, 2018). This functional segregation is supported by neuroanatomical evidence showing largely distinct neural architectures for each modality (Sridhar

Visuospatial working memory, assessed by tasks like the Spatial Span, depends on an integrated fronto-parietal network, with the dorsolateral prefrontal cortex (DLPFC) and posterior parietal cortex (PPC) as central nodes (Barbey et al., 2013). distinct patterns of activation within this network, with the superior parietal lobule primarily supporting spatial storage and the inferior parietal lobule contributing to temporal-spatial

The DLPFC coordinates the maintenance and manipulation of spatial information through top-down executive control signals to posterior parietal regions (Curtis and D’Esposito, 2003; Koenigs et al., 2009). In contrast, auditory-verbal working memory relies more on left-lateralized temporo-frontal circuits, including Broca’s area (Buchsbaum and D’Esposito, 2008).

This neuroanatomical distinction offers a compelling framework for pharmacological predictions. Because the endocannabinoid system, and particularly the CB1 receptors that mediate THC’s effects, are densely distributed throughout cortical association areas including the fronto-parietal network (Rubino and Parolaro, 2011), it is plausible that visuospatial working memory might demonstrate disproportionate vulnerability to THC-mediated disruption compared to auditory-verbal working memory. While some prior meta-analyses have reported greater verbal working memory deficits following cannabis use (Dellazizzo et al., 2022), we hypothesized a specific vulnerability in the visuospatial domain based on the particularly high density of CB1 receptors in the DLPFC and posterior parietal cortex (Glass et al., 1997). We posited that acute THC exposure would disrupt the neural synchrony in these specific fronto-parietal circuits more severely than in the temporo-frontal circuits serving verbal memory.

### Sex as a critical biological variable in cannabis research

Human cognition exhibits well-documented sexual dimorphism. At a population level, males often demonstrate advantages in specific spatial tasks, while females frequently excel in verbal memory domains (Loprinzi and Frith, 2018; Zilles et al., 2016). These differences arise from an interplay of biological and sociocultural factors, with notable variability across tasks and populations (Miller and Halpern, 2014; Voyer et al., 1995). Cannabis use epidemiology reveals complex sex-based patterns. While males have historically

shown higher rates of cannabis use and Cannabis Use Disorder (CUD), this gap is narrowing (Agabio et al., 2016; Bassir Nia et al., 2018; Prieto-Arenas et al., 2022). Furthermore, females often exhibit a “telescoping effect,” an accelerated trajectory from initial use to dependence (Cooper and Craft, 2018; Khan et al., 2013).

The biological underpinnings for these sex differences are robust; the endocannabinoid system is profoundly modulated by gonadal hormones including estradiol and testosterone, which influence CB1 receptor density and signaling in key cortical and limbic regions (Fattore et al., 2014; Gorzalka et al., 2008; Rubino and Parolaro, 2011). Despite this biological foundation, the literature on sex-specific cognitive vulnerability to cannabis remains fragmented and contradictory, with some studies suggesting greater female impairment and others indicating greater male vulnerability (Hasan et al., 2020; Medina et al., 2007; Prieto-Arenas et al., 2022). Many studies, particularly in neuroimaging, have lacked the statistical power or design to properly assess sex as a moderating variable, perpetuating this knowledge gap (Hill et al., 2014; Kroon et al., 2022).

Crucially, these hormonal–endocannabinoid interactions converge on the same fronto-parietal networks that underpin visuospatial working memory. Estradiol downregulates CB1 receptor density in prefrontal and parietal cortices and enhances endogenous anandamide synthesis (Fattore et al., 2014; Gorzalka et al., 2008; Rubino and Parolaro, 2011), potentially attenuating THC’s disruptive effects on these circuits in females. Conversely, the male endocannabinoid milieu—characterized by comparatively higher CB1 receptor availability in these regions—may render the fronto-parietal network more susceptible to acute THC-mediated disruption. This convergence of sex-specific endocannabinoid modulation and network-level vulnerability provides a plausible neurobiological mechanism through which biological sex could moderate the acute cognitive effects of cannabis specifically within the visuospatial domain.

### Rationale and aims of the present study

Given the modality-specific nature of working memory, the established sex differences in endocannabinoid system function, and the contradictory findings in the existing literature, a more nuanced approach is required. Critically, while previous studies have often examined residual effects after periods of abstinence, few have adequately powered investigations of acute intoxication effects with sex as a moderating variable despite the acute state being the precise functional condition during which real-world impairments such as driving errors and workplace accidents occur. The present study was designed to move beyond simplistic main-effects analyses to address this significant gap. The objective was to conduct a systematic, cross-sectional examination of the acute impact of cannabis intoxication on both short-term memory (via Forward span tasks) and working memory (via Backward span tasks). The investigation assessed these effects across both auditory-verbal and visuospatial modalities. A primary aim was to investigate biological sex as a potential moderator of any observed cognitive effects, thereby contributing to a more precise, biologically grounded model of cognitive vulnerability to cannabis.

We hypothesized that acute cannabis intoxication would selectively impair working memory (but not short-term memory) performance, with greater deficits in the visuospatial domain compared to the auditory-verbal domain. Furthermore, based on the neurobiological evidence for sex differences in both spatial cognition and endocannabinoid system function, we predicted that this impairment would be moderated by sex, revealing differential cognitive costs for males versus females. It is important to note that we predicted a differential magnitude of impairment relative to sex-matched controls, not necessarily that males would perform worse than females in absolute terms.

## Materials and methods

### Participants and recruitment

The study sample included 154 adults, comprising 77 individuals who use cannabis regularly and 77 non-using controls. Regular cannabis use was operationally defined as consumption on at least 5 days per week for a minimum of 1 year prior to study enrollment. The sample was balanced for sex, with 78 males and 76 females distributed across the groups. Participant ages ranged from 18 to 50 years (*M* = 24.58, SD = 5.73). Regarding potential neurodevelopmental considerations, while the age range extended to 50 years, the sample was predominantly composed of young adults (mean age approximately 24 years), with the majority within the 18–30 year range. Sensitivity analyses confirmed that the pattern of results remained consistent when restricting analyses to participants aged 18–35 years.

Recruitment utilized a community-based, multi-pronged approach to enhance ecological validity. Participants were approached in informal social settings (e.g., local cafes, public gathering places) across several urban areas. A combination of convenience and snowball sampling was employed, wherein initial participants referred acquaintances, a method suitable for reaching specific user populations. Inclusion criteria for the cannabis group required regular use of a specific, commercially available medical-grade cannabis product. All participants reported no current neurological or psychiatric disorders. Non-using individuals were defined as those reporting fewer than five lifetime cannabis uses and no use within the past year. Exclusion criteria for all participants included a self-reported history of major neurological disorders (e.g., epilepsy, traumatic brain injury) or current/past major psychiatric disorders (e.g., schizophrenia, bipolar disorder), as determined by structured screening interviews.

### Neuropsychological assessment battery demographic and substance use assessment

A comprehensive self-report questionnaire containing 14 items collected demographic information (age, sex) and substance use parameters for participants who use cannabis, including age of initiation and frequency of use. Concurrent use of other substances was also assessed: participants reported their weekly frequency of alcohol consumption and nicotine use. These

variables were not included as covariates in the primary analyses but were retained for a planned sensitivity analysis (see Statistical analysis plan).

### Working memory assessment

The assessment battery consisted exclusively of the four conditions described below (Digit Span Forward, Digit Span Backward, Spatial Span Forward, Spatial Span Backward), selected *a priori* to systematically cross modality (auditory-verbal vs. visuospatial) and task demand (forward/short-term memory vs. backward/working memory) in accordance with the Baddeley-Hitch model. No additional cognitive measures were collected or excluded from reporting.

Short-term and working memory were evaluated using psychometrically validated subtests from the Wechsler Memory Scale-Revised (WMS-R; Wechsler, 1987) and the Wechsler Adult Intelligence Scale-Revised as a Neuropsychological Instrument (WAIS-R NI; Kaplan et al., 1991).

Raw scores were deliberately utilized rather than age-adjusted standard scores to avoid potential cohort effects (e.g., the Flynn Effect; Baxendale, 2010) and to enable direct comparison of absolute performance capacity between groups without normative transformations that might obscure true performance differences. Standard T-scores introduce normative assumptions from standardization samples that may not match the composition of community-recruited research samples, and in the present age-matched design, age-based normative adjustments were not required.

The Digit Span subtest (WMS-R) assessed auditory-verbal memory. In the Forward condition, participants repeat number sequences of increasing length, assessing short-term memory. In the Backward condition, participants repeat the sequences in reverse order. This condition is more cognitively demanding as it requires not only storage but also active mental manipulation of information, thus engaging working memory (Wechsler, 1987). The Spatial Span subtest (WAIS-R NI) assessed visuospatial memory. The apparatus consists of a board with ten irregularly positioned blocks. In the Forward condition, the examiner taps a sequence of blocks and the participant replicates it, assessing visuospatial short-term memory. In the Backward condition, the participant must replicate the sequence in reverse order, a task requiring significant visuospatial working memory resources (Kaplan et al., 1991). For all four conditions, the test was discontinued after two consecutive failures at the same sequence length. Scoring was based on the

number of correctly recalled sequences. Time to complete each task was not recorded, which represents a limitation given previous findings suggesting that reaction time may be sensitive to acute THC effects (Adam et al., 2020).

### Procedure and ethical considerations

The study protocol received approval from the Institutional Ethics Committee of the Western Galilee College (Approval number: WBC-05-017-2023). All participants provided written informed consent. To assess acute cannabis effects, participants in the cannabis group were instructed to consume one standard cigarette (approximately 1 gram) of their own medical-grade cannabis without self-titration. All participants obtained this product from the same licensed supplier, who provides a single, standardized chemovar to users without chronic illness, ensuring a high degree of product homogeneity. The product contained a laboratory-verified cannabinoid profile of 16.1% Δ9-tetrahydrocannabinol (THC) and less than 1% cannabidiol (CBD).

While variations in smoking topography (individual smoking patterns including inhalation depth, breath-hold duration, and puff frequency) represent a methodological consideration, this naturalistic approach enhances ecological validity by reflecting real-world consumption patterns. Moreover, all participants were experienced regular users consuming their typical product, reducing inter-individual variability in achieved effects.

Testing commenced exactly 45 min post-consumption. This window was selected to align with the established pharmacokinetic peak plasma concentration of inhaled THC (Matheson et al., 2020), ensuring assessment occurred during maximal acute influence. All testing was conducted by trained research assistants following standardized administration protocols. The test administration order was fixed across all participants: Digit Span Forward, Digit Span Backward, Spatial Span Forward, Spatial Span Backward.

### Statistical analysis plan

Statistical analyses were performed using SPSS version 28.0 (IBM Corp., Armonk, NY). Statistical significance was set at α = 0.05 for all analyses. Assumptions of normality were assessed using Shapiro-Wilk tests, and homogeneity of variance was tested using Levene’s test. All assumptions were adequately met for the use of parametric analyses. Sample size determination

was conducted *a priori* using G*Power 3.1. For a 2 × 2 between-subjects ANOVA detecting a medium interaction effect (*f* = 0.25), with α = 0.05 and desired power of 0.80, a minimum sample size of 128 participants was required. Our final sample of 154 participants exceeded this requirement, providing power > 0.80 to detect the hypothesized effects. *Post hoc* sensitivity analysis confirmed that our sample size was sufficient to detect effect sizes as small as *f* = 0.23 with 80% power.

Between-group differences in demographic characteristics were examined using one-way ANOVA for age and chi-square tests for categorical variables. Independent samples t-tests were used for cannabis use variables between male and female participants who use cannabis. The primary hypotheses were tested using a series of 2 (Group: Cannabis vs. Control) × 2 (Sex: Male vs. Female) between-subjects ANOVAs for each of the four memory domains. Significant interactions were decomposed using planned *post hoc* independent samples *t*-tests to compare participants who use cannabis to same-sex controls. Although the primary analyses were theory-driven and focused on visuospatial working memory, we additionally applied Bonferroni correction for the four ANOVAs (adjusted α = 0.0125) to ensure statistical robustness. Effect sizes were reported using partial eta-squared (ηp^2^) for ANOVA and Cohen’s d for *t*-tests, interpreted according to Cohen’s (2013) guidelines. It is important to note that the Wechsler Memory Scale subtests are standardized accuracy-based measures; response latency is not recorded. While reaction time measures may capture subtle processing speed effects of THC (Adam et al., 2020), accuracy scores provide a valid and clinically meaningful index of working memory capacity and manipulation ability, reflecting the functional outcome of cognitive processing rather than its temporal dynamics. The extensive psychometric validation literature for the WMS-R and WAIS-R NI demonstrates that accuracy-based span scores reliably index memory capacity, possess strong test-retest reliability (*r* > 0.80), and show convergent validity with neuroimaging measures of prefrontal and parietal function (Kaplan et al., 1991; Wechsler, 1987).

Additionally, to address potential confounding by concurrent substance use, a planned sensitivity analysis was conducted using a 2 (Group) × 2 (Sex) analysis of covariance (ANCOVA) on the primary outcome measure (Spatial Span Backward), with self-reported weekly frequencies of alcohol and nicotine use entered as continuous covariates. This analysis was designed to determine whether the observed effects remained robust after statistically adjusting for these potential confounds.

## Results

### Sample characteristics and group comparability

Demographic and cannabis use characteristics are detailed in Table 1. The four subgroups (male participants who use cannabis, male controls, female participants who use cannabis, female controls) were well-matched for age, with a one-way ANOVA revealing no significant differences, *F*(3, 150) = 0.45, *p* = 0.715. Among participants who use cannabis, males reported a marginally earlier age of onset and higher weekly use frequency compared to females; however, these differences did not achieve statistical significance (*p* = 0.073 and *p* = 0.103, respectively), suggesting comparable chronic use patterns between sexes within the cannabis group.

**TABLE 1:** Participant demographics and cannabis use characteristics.

Variable	Males who use cannabis (*n* = 46)	Male controls (*n* = 32)	Females who use cannabis (*n* = 31)	Female controls (*n* = 45)
Age (years), M (SD)	24.83 (3.31)	25.41 (3.08)	24.16 (3.73)	24.62 (3.12)
Education (years), M (SD)	12.87 (1.41)	13.25 (1.63)	13.03 (1.49)	13.18 (1.47)
Age of onset (years), M (SD)	17.59 (2.35)	—	18.03 (2.48)	—
Duration of use (years), M (SD)	7.24 (3.42)	—	6.13 (3.61)	—
Frequency (days/month), M (SD)	18.93 (8.76)	—	16.48 (9.12)	—

*M*, Mean; *SD*, standard deviation; *F*, F-statistic from one-way ANOVA; *t*, *t*-statistic from independent samples *t*-test; *p*, probability value. Group comparisons for age employed one-way ANOVA; cannabis use characteristics were compared between male and female participants who use cannabis via independent samples *t*-tests. Dashes indicate not applicable.

### Effects on short-term memory and auditory working memory

**FIGURE 1:**
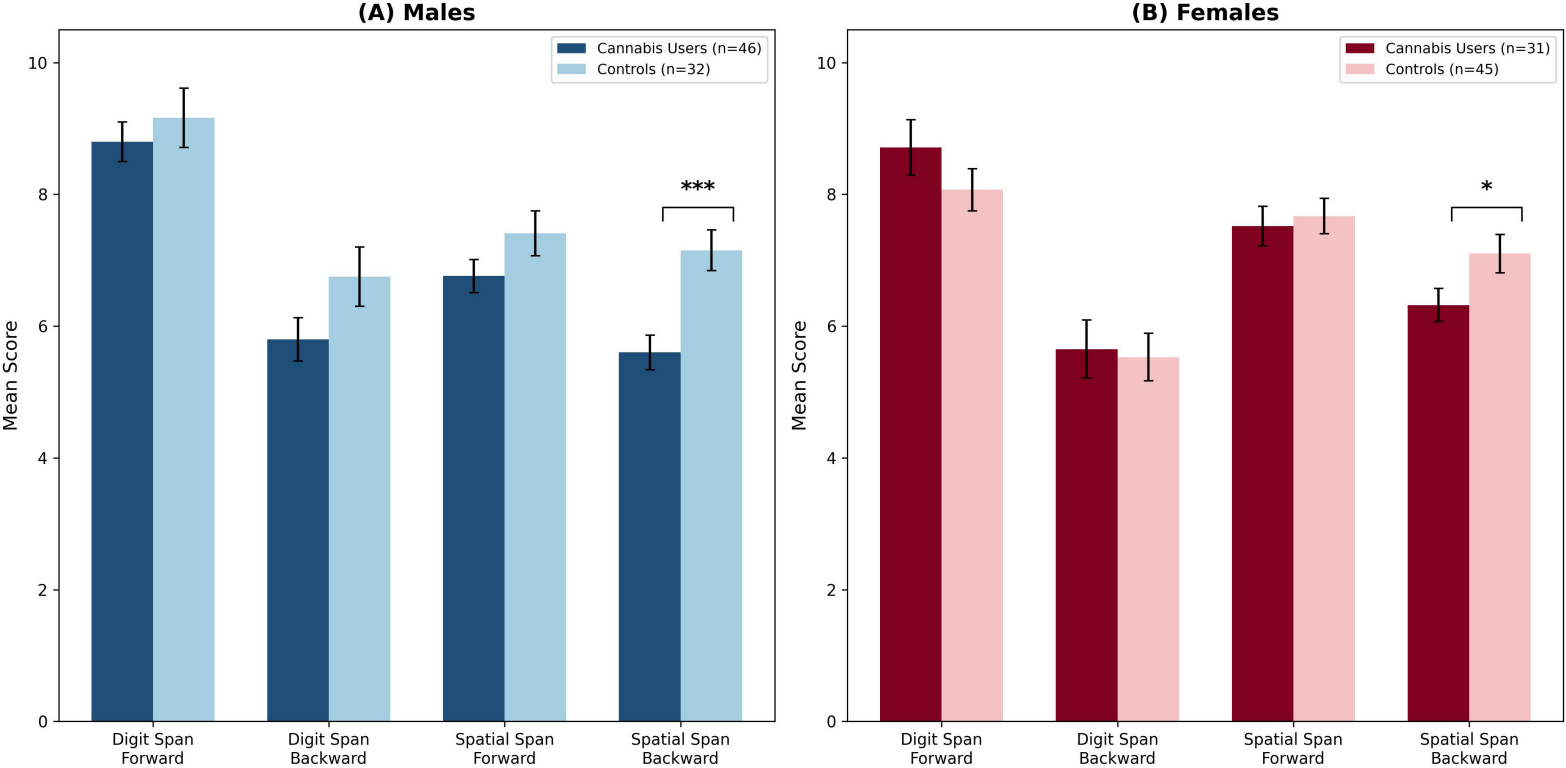
Sex-specific effects of cannabis use on working memory performance. **(A)** Male participants who use cannabis (*n* = 46) compared to male controls (*n* = 32). **(B)** Female participants who use cannabis (*n* = 31) compared to female controls (*n* = 45). Bars represent mean scores with standard error of the mean (SEM). Both panels illustrate the specificity of the cognitive deficit: no significant group differences emerged for auditory short-term memory (Digit Span Forward), auditory working memory (Digit Span Backward), or visuospatial short-term memory (Spatial Span Forward). The significant Group × Sex interaction [*F*(1, 150) = 9.74, *p* < 0.01, η*p*^2^ = 0.061] is evident in the Spatial Span Backward task: both sexes show impairment relative to sex-matched controls, but the magnitude of deficit is significantly larger in males (Cohen’s *d* = –0.87) than in females (Cohen’s *d* = –0.48). Error bars represent ± 1 SEM. *p < 0.05, ***p < 0.001.

To determine the specificity of cannabis-related cognitive effects, performance was first analyzed for the short-term memory and auditory working memory tasks. As shown in Figure 1 and Table 2, the analyses revealed no significant effects of cannabis use in these domains. Specifically, a 2 × 2 ANOVA on Auditory Short-Term Memory (Digit Span Forward) yielded no significant main effect of Group, no main effect of Sex, and no Group × Sex interaction. Within the control group, a non-significant trend was observed where males (*M* = 9.16) performed slightly better than females (*M* = 8.07), *t*(75) = 1.96, *p* = 0.054. Similarly, ANOVAs for Visuospatial Short-Term Memory (Spatial Span Forward) and Auditory Working Memory (Digit Span Backward) also yielded no significant main effects or interactions.

**TABLE 2:** Descriptive statistics for memory task performance by group and sex.

Task	Males who use cannabis (*n* = 46) M (SD)	Male controls (*n* = 32) M (SD)	Females who use cannabis (*n* = 31) M (SD)	Female controls (*n* = 45) M (SD)
Digit Span Forward	8.80 (2.05)	9.16 (2.55)	8.71 (2.33)	8.07 (2.12)
Digit Span Backward	5.80 (2.21)	6.75 (2.52)	5.65 (2.47)	5.53 (2.44)
Spatial Span Forward	6.76 (1.73)	7.41 (1.95)	7.52 (1.67)	7.67 (1.81)
Spatial Span Backward	5.60 (1.77)***	7.15 (1.76)	6.32 (1.40)*	7.10 (1.95)

M = Mean; SD = Standard Deviation. Bold values indicate the task with a significant Group × Sex interaction, *F*(1, 150) = 9.74, *p* < 0.01, η*p*^2^ = 0.061. Asterisks denote significance levels from *post hoc* t-tests comparing participants who use cannabis to same-sex controls: *p < 0.05 (Cohen’s *d* = –0.48, 95% CI [–0.94, –0.02], medium effect), ***p < 0.001 (Cohen’s *d* = –0.87, 95% CI [–1.34, –0.41], large effect). Effect size interpretations follow Cohen (2013).

The absence of significant findings in these three domains indicates that the cognitive impact of acute cannabis intoxication in this sample was not generalized but was highly specific to a particular cognitive function. This null finding across three of four paradigms is consistent with resilience of these cognitive systems to acute intoxication effects, possibly reflecting functional tolerance in this sample of regular users, although reduced task sensitivity cannot be excluded as an alternative explanation.

### Sex-specific effects on visuospatial working memory

In contrast to the other domains, the analysis of visuospatial working memory revealed significant cannabis-related impairment that was moderated by sex. The 2 (Group) × 2 (Sex) between-subjects ANOVA on Spatial Span Backward scores, summarized in Table 3, yielded a significant main effect of Group, *F*(1, 150) = 18.84, *p* < 0.001, ηp^2^ = 0.112, indicating overall impairment in participants who use cannabis. A significant main effect of Sex also emerged, *F*(1, 150) = 4.13, *p* < 0.05, ηp^2^ = 0.027. Critically, these main effects were qualified by a significant Group × Sex interaction, *F*(1, 150) = 9.74, *p* < 0.01, ηp^2^ = 0.061, demonstrating that the effect of cannabis on visuospatial working memory differed significantly between males and females. Crucially, both the main effect of Group and the Group × Sex interaction remained significant under Bonferroni correction (adjusted α = 0.0125), providing robust statistical evidence for cannabis-related visuospatial impairment that is moderated by sex.

**TABLE 3:** Summary of 2 × 2 between-subjects ANOVA for visuospatial working memory performance (Spatial Span Backward).

Source of Variation	*df*	*F*	*p*	Partial η ^2^	90% CI
Group (Cannabis vs. Control)	1	18.84	<0.001***	0.112	[0.04, 0.21]
Sex (Male vs. Female)	1	4.13	<0.05*	0.027	[0.00, 0.10]
Group × Sex	1	9.74	<0.01**	0.061	[0.02, 0.14]
Error	150	–	–	–	–

Dependent variable: Spatial Span Backward raw scores. df, degrees of freedom; , *F*-statistic; *p*, probability value; CI, confidence interval for partial η^2^. *p < 0.05, **p < 0.01, ***p < 0.001. Both the main effect of Group and the Group × Sex interaction remain significant under Bonferroni correction (adjusted α = 0.0125). Dashes indicate not applicable for error term.

*Posst-hoc* decomposition of this interaction confirmed that both male and female participants who use cannabis were significantly impaired relative to their non-using counterparts. Specifically, *post hoc* independent samples *t*-tests revealed that male participants who use

cannabis (*M* = 5.60, SD = 1.77) performed significantly worse than male controls (*M* = 7.15, SD = 1.76), *t*(76) = –3.86, *p* < 0.001, with a large effect size (Cohen’s *d* = –0.87). Female participants who use cannabis (*M* = 6.32, SD = 1.40) also showed significantly impaired performance compared to female controls (*M* = 7.10, SD = 1.95), *t*(74) = –2.10, *p* < 0.05, representing a moderate effect size (Cohen’s *d* = –0.48).

It is essential to clarify the interpretation of these sex differences. The significant Group × Sex interaction indicates that the magnitude of impairment relative to sex-matched controls was significantly greater in males (*d* = –0.87) than in females (*d* = –0.48). This does not imply that male participants who use cannabis performed worse than female participants in absolute terms. Rather, the “cognitive cost” of acute cannabis intoxication on visuospatial manipulation was substantially higher for males than for females when each group is compared to its respective sex-matched baseline. Sensitivity analyses restricting the sample to participants aged 18–35 years (*n* = 138) yielded a consistent pattern of results, with the Group × Sex interaction remaining significant (*p* < 0.05), confirming that findings were not driven by the small number of older participants in the sample.

### Sensitivity analysis: adjusting for concurrent substance use

To address potential confounding effects of concurrent substance use, a sensitivity analysis was conducted utilizing a 2 (Group) × 2 (Sex) analysis of covariance (ANCOVA) on the Spatial Span Backward scores, with self-reported weekly frequencies of alcohol and nicotine use entered as continuous covariates. Preliminary assumption checks supported the use of ANCOVA, with no evidence of heterogeneous regression slopes (covariate × Group and covariate × Sex interaction terms were non-significant).

Neither covariate significantly predicted visuospatial working memory performance (Alcohol: *F*(1, 148) = 1.12, *p* = 0.291, ηp^2^ = 0.008; Nicotine: *F*(1,148) = 0.85, *p* = 0.358, ηp^2^ = 0.006). Crucially, the adjusted model fully replicated the core pattern observed in the primary analyses. The main effects of Group [*F*(1, 148) = 17.56, *p* < 0.001, ηp^2^ = 0.106] and Sex [*F*(1, 148) = 3.98, *p* = 0.048, ηp^2^ = 0.026] remained significant. Most importantly, the critical Group × Sex interaction retained its statistical significance [*F*(1, 148) = 8.23, *p* = 0.005, ηp^2^ = 0.053], successfully surviving the Bonferroni-corrected threshold (α = 0.0125). These results demonstrate that the disproportionate visuospatial working memory impairment observed in males relative to same-sex controls is robust to statistical adjustment for concurrent alcohol and nicotine use.

## Discussion

### Principal findings: a sex-moderated, modality-specific deficit

The present work provides evidence that the neurocognitive impact of acute cannabis intoxication is not uniform but is instead highly specific and moderated by biological sex. The central finding is that acute cannabis intoxication was associated with selective impairment of visuospatial working memory, while auditory-verbal and short-term memory domains remained unaffected. Crucially, this deficit was present in both males and females, but the magnitude of impairment relative to same-sex controls was significantly larger in males (a large effect, *d* = –0.87) compared to females (a moderate effect, *d* = –0.48). This finding of heightened male vulnerability relative to baseline, situated within a context of impairment in both sexes, contributes to our understanding of cannabis neuropharmacology. It highlights the necessity of considering interactions between drug exposure, cognitive modality, and individual biological factors.

Notably, the absence of significant between-group differences in three of four cognitive paradigms represents an important finding in itself. This pattern suggests that individuals who use cannabis regularly may demonstrate resilience to acute intoxication effects across multiple working memory systems, with the selective exception of visuospatial manipulation. One plausible account is that tolerance develops differentially across cognitive systems as a function of regional CB1 receptor density, with circuits subserving auditory-verbal memory and short-term storage developing more complete tolerance than the densely CB1-innervated fronto-parietal network. However, we acknowledge that it is equally possible that the three unaffected tasks were not sufficiently sensitive to detect subtle impairments, and this alternative cannot be excluded without more fine-grained measures (e.g., reaction time, adaptive difficulty paradigms).

### Neurobiological mechanisms and sex-specific vulnerability

The specificity of the observed deficits implicates targeted disruption of the fronto-parietal neural network. The Spatial Span Backward task is a well-validated probe of the visuospatial sketchpad, a system critically dependent on the integrity of the DLPFC and PPC (Barbey et al., 2013; Seo et al., 2012). While CB1 receptors are widely distributed throughout the brain, including

high densities in the basal ganglia and cerebellum, our interpretation focuses on the cortical density within the prefrontal cortex and parietal lobes. These specific regions constitute the core substrate of the visuospatial sketchpad. The acute disruption observed specifically in the backward spatial task aligns with THC-induced dysregulation in these fronto-parietal circuits rather than motor coordination regions.

Convergent support for this interpretation emerges from neuroimaging literature, which reveals altered brain activation in individuals who use cannabis during working memory tasks. These alterations range from hypoactivation to compensatory hyperactivation, where individuals recruit additional neural resources to maintain performance (Kanayama et al., 2004; Schweinsburg et al., 2008). Specifically, meta-analytic evidence points to consistent hypoactivation in the DLPFC (Yanes et al., 2018), a finding complemented by studies showing weaker neural oscillations in both the DLPFC and ventrolateral prefrontal cortex during working memory tasks (Huang et al., 2025).

Conversely, compensatory hyperactivation is often observed in other regions, including the parietal cortex (Schweinsburg et al., 2008), anterior cingulate cortex (Kanayama et al., 2004), and striatum (Ma et al., 2018). Notably, these patterns of altered neural activity frequently occur even when individuals who use cannabis maintain behavioral performance comparable to controls (Chang et al., 2006; Kanayama et al., 2004; Padula et al., 2007).

This dissociation between neural effort and behavioral output is widely interpreted as evidence of fundamental neural inefficiency. Speculatively, the pronounced behavioral deficits observed in our male participants (Cohen’s *d* = –0.87) could represent a failure point of these compensatory neural mechanisms—a threshold beyond which the brain’s capacity to recruit additional resources is exhausted; however, this interpretation requires direct neuroimaging validation. Under acute THC intoxication, males may reach this compensatory ceiling more readily than females, resulting in observable performance decrements. This interpretation suggests that while both sexes engage compensatory strategies, male neural systems may operate closer to their compensatory limits, leaving less reserve capacity to buffer against THC-induced disruption.

Regarding sex-specific vulnerability, it is essential to refine interpretation of the observed differences. Our data do not imply that males who use cannabis invariably perform worse than females in absolute terms. Rather, the significant Group × Sex interaction reveals a greater magnitude of impairment in males relative to their sex-matched baseline. This pattern suggests that females may possess neurobiological resilience factors that buffer the acute disruptive effects of THC on fronto-parietal networks.

As a *post hoc* interpretive framework requiring future empirical validation, we note that estradiol may exert neuroprotective effects on the endocannabinoid system through multiple converging mechanisms: (a) it downregulates CB1 receptor density in key cortical regions including the prefrontal cortex, potentially reducing the number of available targets for THC binding; (b) it enhances the synthesis of anandamide (AEA), the primary endogenous cannabinoid, which may compete with exogenous THC at receptor sites; and (c) it modulates fatty acid amide hydrolase (FAAH) activity, thereby influencing overall endocannabinoid tone and receptor availability (Fattore et al., 2014; Gorzalka et al., 2008; Rubino and Parolaro, 2011).

These estrogen-mediated effects collectively may attenuate THC’s ability to disrupt neural signaling in the prefrontal and parietal cortices, providing females with a hormonal “buffer” against acute cognitive disruption. Additionally, sex differences in the activity of hepatic cytochrome P450 enzymes (particularly CYP3A4) may contribute to differential THC metabolism and brain exposure, with females potentially achieving lower peak brain THC concentrations for equivalent doses (Craft et al., 2014; Reich et al., 2009).

Future research incorporating hormonal assays, particularly measurements of circulating estradiol levels across the menstrual cycle, and pharmacokinetic measures of plasma THC concentrations would help validate these hypothesized protective mechanisms.

### Functional implications

The observed deficit in visuospatial working memory (VSWM) has potential implications for functional capacity in several real-world domains. VSWM supports tasks requiring spatial navigation, distance judgment, and the maintenance of spatial relationships in working memory (Johannsdottir and Herdman, 2010). The link between VSWM and driving performance has been established in the literature (Johannsdottir and Herdman, 2010), and cannabis use has been associated with impaired driving in controlled studies (Hartman and Huestis, 2013; Marcotte et al., 2022). Our findings suggest a potential neurocognitive mechanism contributing to this association. However, we emphasize that the current study did not directly assess driving performance or other functional outcomes, and extrapolations to real-world behavior should be made cautiously pending validation studies.

Similarly, VSWM is relevant to performance in skilled occupations requiring spatial planning and motor coordination (Bo et al., 2009; Montgomery et al., 2010). The observed pattern of sex-specific vulnerability may warrant consideration in the development of targeted harm-reduction messaging, though specific policy recommendations would require additional research establishing direct links between these laboratory findings and occupational outcomes.

From a translational perspective, these findings have direct implications for precision medicine approaches to cannabis therapeutics and risk assessment. As medical cannabis use expands globally for conditions ranging from chronic pain to epilepsy, understanding individual difference factors that predict cognitive vulnerability becomes clinically essential. The demonstration that biological sex moderates cannabis-induced cognitive impairment with males showing nearly double the effect magnitude (*d* = –0.87 vs. *d* = –0.48) provides an empirical foundation for sex-informed clinical guidelines. Specifically, these data support the development of: (a) sex-differentiated dosing recommendations for medical cannabis, particularly for formulations intended for use during activities requiring visuospatial processing; (b) sex-specific safety thresholds for activities requiring intact spatial cognition, such as operating motor vehicles or heavy machinery; and (c) targeted harm-reduction messaging that acknowledges differential vulnerability patterns. Future clinical frameworks should incorporate biological sex as a key variable in cannabis risk stratification algorithms, thereby advancing toward truly personalized approaches in cannabis-related healthcare policy. The magnitude of the sex difference observed (males showing 81% greater impairment than females relative to same-sex controls) is clinically meaningful and warrants integration into both clinical practice and public health communications.

### Strengths and limitations

This study has several strengths, including a multi-modal memory assessment that revealed a highly specific cognitive deficit, direct statistical testing of sex as a moderating variable with adequate statistical power, and the use of a standardized, commercially available cannabis product that enhances ecological validity. The *a priori* power analysis and subsequent confirmation of adequate statistical power strengthen confidence in our findings. The use of convenience and snowball sampling in naturalistic settings, while limiting strict generalizability, enhances ecological validity by reflecting real-world cannabis use contexts.

However, several limitations must be acknowledged. First, the cross-sectional design establishes an association but cannot prove a causal link between cannabis use and cognitive deficits; pre-existing differences cannot be ruled out. Second, a significant limitation is the inability to fully disentangle whether the observed deficits are attributable to the acute intoxicating effects of cannabis, the long-term effects of chronic use, or an interaction of both. The current design captures the “cumulative functional reality” of the regular user under acute intoxication, but future studies with within-subjects designs or first-time users would help address this confound.

Third, a significant limitation is the absence of biological verification of substance use status. No biological samples (e.g., saliva or urine) were collected to objectively verify THC exposure levels in the cannabis group or to confirm abstinence in the control group. While all participants were systematically screened via self-report, the lack of toxicological confirmation means that individual variation in actual THC bioavailability and the possibility of undisclosed substance use in controls cannot be excluded. Future studies should incorporate toxicology panels to strengthen internal validity.

Fourth, the study did not utilize a structured clinical interview (e.g., SCID) for psychiatric diagnosis, limiting our ability to completely rule out all potential comorbidities, although self-reports were systematically screened.

Fifth, while the dose was standardized in quantity (one cigarette, approximately 1 g), variability in smoking topography (e.g., inhalation depth, breath-hold duration) could not be controlled. However, such variability would be expected to add statistical noise rather than systematic bias, potentially attenuating rather than inflating effect sizes. That robust sex-specific effects emerged despite this methodological noise strengthens confidence in the findings.

Sixth, the study did not systematically control for potential covariates such as education level or IQ. However, although concurrent alcohol and nicotine use was not included in the primary models, we explicitly addressed this via a sensitivity ANCOVA controlling for weekly alcohol and nicotine consumption frequency. The Group × Sex interaction on Spatial Span Backward remained robustly significant [*F*(1, 148) = 8.23, *p* = 0.005, ηp^2^ = 0.053], mitigating concerns that concurrent substance use accounts for the primary visuospatial deficit. Furthermore, several other factors strongly mitigate concerns about confounding: (a) the four subgroups were well-matched for age; (b) the domain-specificity of findings—impairment in only one of four tasks—is inconsistent with a generalized confounding variable; and (c) the observed effect sizes substantially exceed those typically attributable to uncontrolled confounds in well-matched community samples. Nonetheless, the absence of direct measures of education level and cognitive ability (e.g., estimated IQ) remains a limitation; future studies should directly control for these variables to further strengthen internal validity. Additionally, no data on age of onset of regular use (only age of first use) or task completion times were collected. These limitations should be addressed in future research.

### Future directions and conclusion

The findings from this study lay groundwork for a more nuanced understanding of cannabis’s cognitive effects and point toward critical avenues for future research. Longitudinal studies that assess cognition before and after cannabis initiation are needed to establish causality definitively (Meier et al., 2012).

Placebo-controlled laboratory studies using standardized THC doses would allow for isolation of specific cannabinoid effects and examination of dose-response relationships. The most crucial next step is to integrate these behavioral findings with neurobiological measures. Future studies combining neuroimaging with hormonal and genetic profiling are needed to validate and extend these sex-specific mechanisms. Such work could directly test the neural strategy hypothesis by comparing brain activation patterns in males and females during visuospatial tasks and could validate hormonal hypotheses by correlating circulating hormone levels with cognitive performance.

This study makes three contributions to the cannabis literature: (1) it demonstrates that cannabis-related cognitive deficits show remarkable specificity to visuospatial working memory while sparing other memory domains; (2) it provides direct statistical evidence that biological sex moderates this effect, with males showing nearly twice the impairment magnitude relative to their sex-matched baseline compared to females; and (3) it links these findings to potential real-world functional implications including driving safety and occupational performance.

These insights build upon previous work that either examined sex differences without adequate statistical power or assessed cognition without considering modality-specific effects.

In conclusion, this research demonstrates that the cognitive impact of cannabis is not a simple, uniform phenomenon. It reflects a complex interplay between the drug’s pharmacology, the specific cognitive system being taxed, and the biological sex of the individual. The central finding that acute cannabis intoxication impairs visuospatial working memory in both sexes, but to a significantly greater degree in males relative to their sex-matched baseline (81% greater effect magnitude), supports a more sophisticated, sex-conscious approach in both scientific research and public health communication. These findings provide an empirical foundation for precision medicine approaches to cannabis therapeutics, suggesting that biological sex should be incorporated as a key variable in clinical risk assessment and dosing guidelines. By revealing that biological sex is a critical moderator of cannabis’s neurocognitive impact, this work advances the field toward more precise, individualized models of risk and vulnerability.

## Data Availability

The raw data supporting the conclusions of this article will be made available by the authors, without undue reservation.
